# An Inflammation-Adjusted Framework for Nutritional Assessment in Multimorbid Patients with Advanced Neurological Diseases

**DOI:** 10.3390/medicina62081587

**Published:** 2026-08-18

**Authors:** Viljaras Reigas, Deimantas Terleckij, Ingrida Šukienė

**Affiliations:** Nursing Department, SMK College of Applied Science, 91199 Klaipeda, Lithuania

**Keywords:** advanced neurological diseases, malnutrition, inflammation, laboratory nutritional biomarkers, enteral nutrition, palliative care, albumin, transferrin, clinical nutrition

## Abstract

*Background and Objectives:* Laboratory parameters associated with nutritional and metabolic status may be substantially influenced by inflammatory activity, complicating their interpretation in patients with advanced neurological diseases. This study aimed to characterize within-patient changes in inflammatory biomarkers and laboratory parameters related to nutritional and metabolic status in multimorbid patients receiving long-term enteral nutrition, with particular emphasis on differences between infectious and clinically non-infectious periods. A secondary objective was to propose a conceptual framework for interpreting these laboratory parameters in the context of inflammatory activity. *Materials and Methods:* A prospective longitudinal observational case series was conducted in an inpatient palliative care facility. Of 23 patients who entered prospective follow-up, five completed the predefined nine-month observation period and were included in the final longitudinal analysis. Twenty-three predefined assessment time points were evaluated for each participant. Inflammatory parameters, serum proteins, micronutrient-related measurements, and vitamin concentrations were analyzed descriptively in relation to the clinical course of each patient. *Results:* Patients with recurrent infectious episodes demonstrated pronounced increases in inflammatory biomarkers accompanied by decreases in serum proteins, particularly albumin, transferrin, and total protein, as well as changes in serum iron (Fe) and zinc (Zn). The greatest fluctuations were observed in patients with the highest inflammatory burden, whereas patients without recurrent infections showed comparatively stable laboratory profiles. Vitamin A, vitamin D, and folate concentrations showed relatively limited fluctuations. Importantly, all five patients received continued enteral nutritional support, with daily energy intake ranging from 1450 to 1800 kcal, and body weight increased by 1.0–3.0 kg during follow-up. Based on these case-based observations and current evidence, a conceptual framework for interpreting laboratory parameters in the context of inflammatory activity was proposed. *Conclusions:* In patients with advanced neurological diseases, laboratory parameters associated with nutritional and metabolic status should be interpreted in the context of inflammatory activity and should not be used independently to determine nutritional status. The proposed conceptual framework is intended as an interpretative aid for laboratory findings rather than as an alternative diagnostic system for malnutrition and requires validation in larger prospective studies.

## 1. Introduction

Malnutrition is a common and clinically significant complication in patients with advanced neurological disorders [1]. Progressive neurological conditions frequently result in dysphagia, reduced physical activity, cognitive decline, complete dependence on nursing care, and the need for long-term enteral nutrition [2,3,4]. Malnutrition in this population is multifactorial, reflecting not only inadequate nutritional intake but also sarcopenia, altered energy expenditure, recurrent infections, and chronic inflammation; consequently, even long-term enteral nutrition may not be sufficient to maintain adequate nutritional status [5,6,7].

Current nutritional assessment frameworks, including those proposed by GLIM, ESPEN, and ASPEN, recognize the limitations of laboratory biomarkers in the diagnosis of malnutrition. In the GLIM framework, inflammation is considered an etiologic rather than a phenotypic criterion, and measurement of inflammatory biomarkers is not routinely required for the diagnosis of malnutrition [8,9,10,11]. Accordingly, laboratory and inflammatory biomarkers should not be regarded as standalone diagnostic criteria for malnutrition.

Systemic inflammation may substantially influence laboratory parameters commonly encountered in nutritional assessment. The acute-phase response increases positive acute-phase proteins such as CRP while reducing negative acute-phase proteins, including albumin and transferrin [12,13,14,15,16,17,18,19]. Inflammation may also alter circulating micronutrient concentrations through micronutrient-specific mechanisms; for example, hepcidin-mediated changes reduce circulating Fe, whereas serum Zn may decrease through inflammation-associated redistribution [20,21,22]. These effects complicate the interpretation of laboratory findings during inflammatory activity.

Although contemporary nutritional assessment frameworks appropriately limit the diagnostic role of laboratory biomarkers, these parameters remain frequently measured in clinical practice. Their interpretation may be particularly challenging in patients with advanced neurological disorders, in whom malnutrition and inflammation frequently coexist. Previous studies have primarily focused on the biological or prognostic significance of individual biomarkers [23,24,25], whereas evidence regarding their longitudinal behavior in this specific population remains limited.

Both acute and persistent inflammation may affect laboratory parameters, although their clinical and biochemical patterns differ [26]. Acute inflammatory episodes are generally readily identifiable, whereas persistent low-grade inflammation may occur without overt infection or pronounced elevations in conventional inflammatory markers [27,28,29,30,31,32,33,34,35,36]. Consequently, persistently altered laboratory values may be difficult to distinguish from changes associated with nutritional deterioration when interpreted without the broader clinical and inflammatory context [37,38,39].

Therefore, this prospective longitudinal case series aimed to characterize within-patient changes in inflammatory biomarkers and laboratory parameters related to nutritional and metabolic status in multimorbid patients with advanced neurological diseases receiving long-term enteral nutrition, with particular emphasis on differences between infectious and clinically non-infectious periods. Based on these case-based observations and current evidence, a secondary objective was to propose a conceptual framework for interpreting these laboratory parameters in the context of inflammatory activity, without positioning them as diagnostic criteria for malnutrition.

## 2. Materials and Methods

### 2.1. Study Design and Setting

The study was conducted at an inpatient palliative care facility. The observation period lasted from July 2025 to April 2026. A nine-month follow-up period was chosen to provide a standardized observation interval for all participants while covering the longest hospitalization period among the study patients.

A prospective longitudinal observational study based on a case series design was conducted in multimorbid patients with advanced neurological diseases. At the beginning of the study, 23 patients met the predefined eligibility criteria and entered prospective follow-up. During the observation period, 12 patients died, four were transferred to other hospitals, and two were discharged home, after which follow-up at the study facility was no longer possible. Consequently, these 18 patients did not complete the predefined longitudinal observation period and did not have complete data across the scheduled assessment time points. The final longitudinal analysis therefore included five patients who completed the observation period and had data available throughout the predefined follow-up.

### 2.2. Study Population and Eligibility Criteria

Patients were eligible for inclusion if they met all of the following criteria: (1) advanced neurological disease associated with complete dependence on nursing care, including bedridden status, severe functional impairment, cognitive impairment, involuntary movements, dystonic episodes, or progressive neurological deterioration; (2) a Barthel Index score of 0, indicating complete functional dependence; (3) long-term enteral nutrition administered via percutaneous endoscopic gastrostomy (PEG) or a nasogastric tube (NG); (4) low body mass index (BMI), indicating undernutrition; (5) the presence of stage II–IV pressure injuries according to the European Pressure Ulcer Advisory Panel/National Pressure Injury Advisory Panel (EPUAP/NPIAP) classification; (6) no disease-modifying treatment for the underlying neurological disorder; and (7) written informed consent provided by the patient’s legally authorized representative.

Eligibility was determined prospectively at study enrollment according to the predefined inclusion and exclusion criteria described above. Only patients meeting all inclusion criteria were enrolled in the study. For the final longitudinal analysis, participants were required to complete the predefined observation period and have data available across the scheduled assessment time points. Follow-up discontinuation due to death, transfer to another healthcare facility, or discharge home resulted in exclusion from the final longitudinal analysis because subsequent scheduled assessments could no longer be performed.

**Figure 1:**
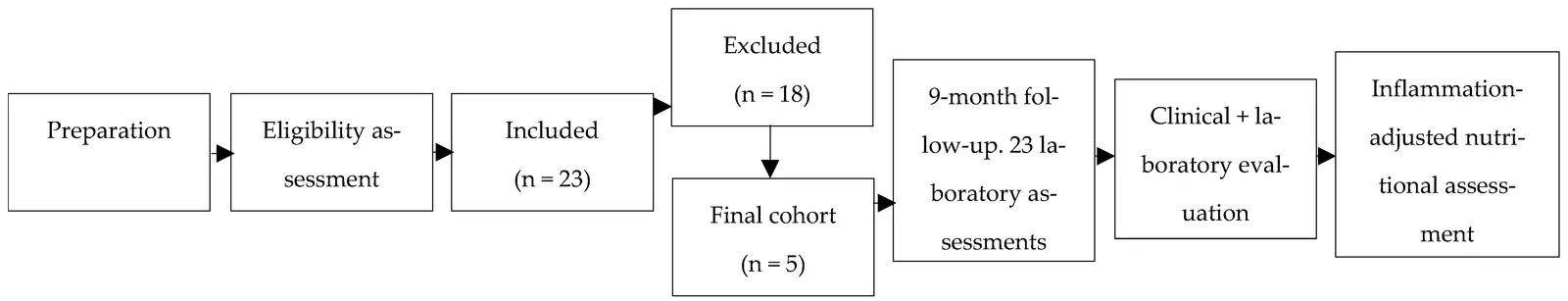
Study Flow.

The study flow, including participant eligibility, follow-up, reasons for discontinuation, and inclusion in the final longitudinal analysis, is presented in Figure 1.

### 2.3. Data Collection and Laboratory Assessment

During the study, data were prospectively recorded using a study-specific case report form developed by the investigators. The following information was collected for each participant: demographic characteristics, anthropometric measurements, primary neurological diagnosis, clinical characteristics of neurological impairment, duration of hospitalization, duration of palliative care, route of enteral nutrition, current treatment, dates of laboratory testing, and corresponding laboratory results.

Predefined assessment time points were established to standardize the longitudinal analysis of laboratory biomarkers across all participants. Blood samples were collected at each of the 23 scheduled assessment time points. If sample collection was not feasible on the scheduled date because of the patient’s clinical condition, blood sampling was performed on the nearest possible day. All laboratory analyses were performed in the same accredited clinical laboratory using standardized analytical methods and quality-controlled procedures.

The laboratory investigations were grouped according to their primary interpretative domain. Inflammatory parameters included CRP, procalcitonin, total leukocyte count, absolute and relative neutrophil counts, ESR, IL-6, TNF-α, and hepcidin. Serum proteins included albumin, total protein, and transferrin. Micronutrient-related measurements included serum selenium (Se), Fe, and Zn, while vitamin status was assessed using vitamin A, vitamin D, and folate concentrations. These groups were analyzed separately because their biological responses to inflammation and their clinical interpretability differ. None of these laboratory parameters were used as standalone diagnostic criteria for malnutrition.

### 2.4. Outcomes and Statistical Analysis

Because of the small sample size, inferential statistical analyses were not performed. The study findings are therefore presented using a descriptive analytical approach, focusing on the longitudinal changes in laboratory biomarkers and their interpretation within the clinical context of each individual patient. The primary outcome of the study was the temporal relationship between inflammatory biomarkers and laboratory parameters related to nutritional and metabolic status throughout the observation period.

### 2.5. Ethical Considerations

The study was conducted in accordance with the fundamental principles of biomedical research ethics, including respect for participant autonomy, confidentiality, beneficence, non-maleficence, and assessment of the balance between potential benefits and risks. Because all participants had advanced neurological impairment and lacked decision-making capacity, written informed consent was obtained from each patient’s legally authorized representative prior to study participation.

The study protocol was approved by the SMK University of Applied Sciences Bioethics Committee (Protocol No. 14, 18 April 2025). The study was conducted in accordance with the ethical principles of the Declaration of Helsinki for Medical Research Involving Human Subjects. All procedures involving personal data complied with the requirements of the European Union General Data Protection Regulation (GDPR; Regulation (EU) 2016/679). Participant data were anonymized prior to analysis and used exclusively for scientific research purposes.

## 3. Results

The final analytical cohort comprised five patients with different advanced neurological diseases. Participants ranged in age from 18 to 82 years. All patients were completely dependent on nursing care, received long-term enteral nutrition, and were under inpatient palliative care. The duration of palliative care ranged from 4 to 27 months. The demographic and clinical characteristics of the study participants are summarized in Table 1.

All five patients received continuous long-term enteral nutritional support throughout the observation period, with daily energy intake ranging from 1450 to 1800 kcal. Despite low baseline BMI values, body weight increased in all participants during follow-up. Weight gain ranged from 1.0 to 3.0 kg, with the greatest increase observed in Patient D (+3.0 kg), while Patients A, B, C, and E gained 2.3, 1.0, 2.0, and 1.0 kg, respectively.

The principal clinical characteristics associated with chronic malnutrition risk were evaluated in all participants. Variables included phenotypic indicators of malnutrition, severity of neurological impairment, inflammatory status, comorbidities, frequency of infectious episodes, and other clinical conditions potentially influencing the interpretation of laboratory parameters related to nutritional and metabolic status. The distribution of these characteristics is summarized in Table 2.

All participants exhibited clinical characteristics consistent with a high risk of malnutrition. Advanced neurological disease, severe functional impairment, dysphagia, low body mass index, immobility, and long-term enteral nutrition were common to all patients. Four of the five participants had sarcopenia, and multimorbidity was present in the entire cohort. Collectively, these findings indicate that all patients exhibited multiple phenotypic and etiological factors associated with malnutrition and represented a population at particularly high nutritional risk.

Recurrent infectious episodes were documented in three patients (A–C), with two to four episodes recorded during the preceding three months. Infection was confirmed by both clinical and microbiological findings in one patient and by clinical assessment alone in two patients. Four participants had stage II–IV pressure injuries, indicating a substantial burden of chronic tissue injury within the study cohort.

Analysis of comorbidities showed that all participants had gastrointestinal disorders potentially affecting nutrient intake and/or absorption, as well as cognitive impairment and psychiatric disorders. None of the patients had a diagnosed malignancy. Chronic heart failure was present in two patients, whereas chronic kidney disease was identified in one participant.

Inflammatory biomarker profiles were evaluated in all participants to characterize inflammatory activity throughout the observation period. The analysis included conventional inflammatory biomarkers, namely, CRP, procalcitonin, total leukocyte count, absolute and relative neutrophil counts, and ESR, together with the pro-inflammatory cytokines IL-6 and TNF-α. To facilitate comparison between inflammatory and non-inflammatory states, the minimum and maximum values of each biomarker are presented for periods of confirmed infection (Y) and periods without clinical evidence of infection (N) (Table 3).

Marked fluctuations in inflammatory biomarkers were observed in the three patients (A–C) who experienced recurrent infectious episodes during the observation period. During infectious episodes, all patients demonstrated increased concentrations of CRP, procalcitonin, leukocyte count, neutrophils, ESR, and IL-6, while elevated TNF-α concentrations were observed in some patients. The most pronounced inflammatory response was recorded in Patient A, who exhibited a peak CRP concentration of 274.67 mg/L, a procalcitonin concentration of 1.450 ng/mL, and an IL-6 concentration of 120.0 pg/mL.

During periods without clinical evidence of infection, the concentrations of most inflammatory biomarkers decreased; however, complete normalization was not observed in all patients. Patients A, B, C, and E exhibited persistently elevated ESR, while Patient E also demonstrated mildly but consistently elevated CRP concentrations throughout the observation period. These findings indicate that inflammatory activity persisted in some participants despite the absence of clinically apparent infectious episodes.

No infectious episodes were documented in Patient D during the observation period. Accordingly, inflammatory biomarkers remained stable and were consistently within or close to the reference range throughout follow-up. In contrast to patients with recurrent infections, no marked fluctuations in inflammatory biomarker concentrations were observed.

Overall, the study cohort demonstrated considerable heterogeneity in inflammatory activity, ranging from stable inflammatory biomarker profiles to recurrent acute infectious episodes accompanied by marked increases in inflammatory biomarkers. These findings provide the basis for subsequent evaluation of the relationship between inflammatory activity and laboratory parameters related to nutritional and metabolic status.

Laboratory biomarkers related to nutritional and metabolic status were analyzed during periods of confirmed infection (Y) and periods without clinical evidence of infection (N). The analysis included serum proteins (albumin, total protein, and transferrin), selected micronutrient-related measurements (Se, Fe, and Zn), and vitamins (vitamin A, vitamin D, and folate). The minimum and maximum values of these biomarkers for each patient are presented in Table 4.

Patients A, B, and C, who experienced recurrent infectious episodes during the observation period, exhibited lower concentrations of the measured serum proteins (albumin, total protein, and transferrin) during infectious periods compared with periods without clinical evidence of infection. The most pronounced changes in serum protein concentrations were observed in Patients A and C, who also demonstrated the highest inflammatory biomarker concentrations. Serum Fe and Zn concentrations also tended to be lower during infectious periods; however, these micronutrient-related measurements were considered separately because their circulating concentrations may be influenced by inflammation through mechanisms that differ from those affecting serum proteins.

In contrast, no infectious episodes were documented in Patient D during the observation period, and laboratory biomarkers related to nutritional and metabolic status remained stable and within or close to the reference range. Patient E exhibited only minor fluctuations in albumin, transferrin, and total protein concentrations, while vitamin A, vitamin D, and folate concentrations remained relatively stable throughout the observation period.

Vitamin A, vitamin D, and folate concentrations showed comparatively limited fluctuations between infectious and non-infectious periods in the present case series. This pattern differed from the more pronounced changes observed in serum proteins and selected micronutrient-related measurements; however, given the small sample size and the micronutrient-specific effects of inflammation, these observations should not be interpreted as evidence that vitamin concentrations are generally unaffected by inflammatory activity.

To visualize the temporal relationship between inflammatory activity and laboratory biomarkers related to nutritional and metabolic status, longitudinal graphs were constructed for the principal biomarkers. The figures present changes in CRP, albumin, transferrin, and total protein concentrations across 23 predefined assessment time points for all participants. Red arrows indicate peaks corresponding to clinically and/or microbiologically confirmed infectious episodes.

**Figure 2:**
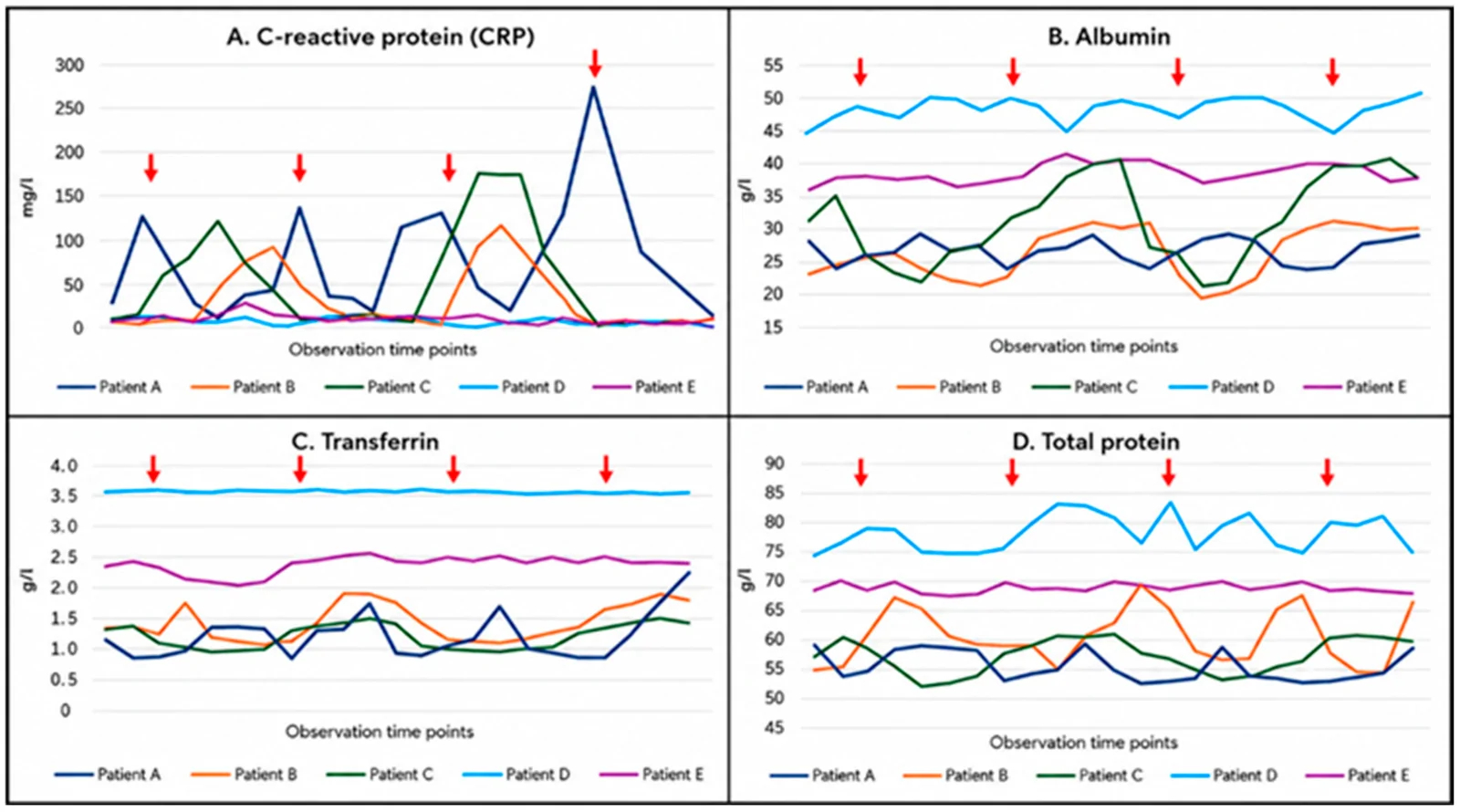
Longitudinal changes in inflammatory and nutritional biomarkers during the observation period. Red arrows indicate peaks of infectious episodes.

The longitudinal graphs (Figure 2) demonstrate that infectious episodes were accompanied by pronounced increases in CRP concentrations in most participants, which coincided with decreases in albumin, transferrin, and total protein concentrations. The most pronounced fluctuations in inflammatory biomarkers and laboratory parameters related to nutritional and metabolic status were observed in Patients A and C, who experienced the most frequent infectious episodes. In contrast, Patient D, who had no documented infectious episodes, maintained relatively stable inflammatory biomarkers and laboratory parameters throughout the observation period. Patient E demonstrated only minor fluctuations in inflammatory biomarkers, with correspondingly smaller changes in albumin, transferrin, and total protein concentrations.

Longitudinal analysis of individual patient profiles demonstrated a temporal association between inflammatory activity and changes in the measured laboratory parameters. In most cases, peak CRP concentrations coincided with the lowest albumin, transferrin, and total protein concentrations, whereas subsequent decreases in CRP were accompanied by increases in these parameters. This temporal pattern was most pronounced in patients who experienced recurrent infectious episodes.

## 4. Discussion

The principal finding of the present case series was a consistent temporal association between inflammatory activity and changes in several laboratory parameters related to nutritional and metabolic status in patients with advanced neurological diseases receiving long-term enteral nutrition. Across individual patient trajectories, increases in inflammatory biomarkers frequently coincided with decreases in albumin, transferrin, total protein, serum Fe, and Zn. These observations suggest that changes in these laboratory parameters should be interpreted cautiously during periods of inflammatory activity and should not be considered in isolation as evidence of deterioration in nutritional status.

Although the influence of inflammation on these laboratory parameters is well established [40,41], the present case series illustrates this relationship through repeated longitudinal observations within individual patients. This distinction is particularly relevant in patients with advanced neurological disease, in whom long-term enteral nutrition, severe functional impairment, recurrent infections, pressure injuries, and other comorbid conditions may coexist. Rather than establishing new diagnostic biomarkers of malnutrition, the present findings demonstrate how the interpretation of routinely available laboratory measurements may change according to the inflammatory context.

This pattern was particularly evident in Patients A–C, who experienced recurrent infectious episodes during follow-up. Periods of increased inflammatory activity were accompanied by lower concentrations of albumin, transferrin, total protein, serum Fe, and Zn, with the most pronounced fluctuations observed in Patients A and C. In contrast, Patient D, in whom no infectious episodes were documented, showed comparatively stable inflammatory and laboratory profiles. Patient E demonstrated smaller fluctuations in inflammatory activity and correspondingly less pronounced changes in serum protein concentrations. Although causal relationships cannot be established from this small observational case series, these contrasting individual trajectories support the relevance of considering inflammatory status when interpreting longitudinal laboratory results.

Importantly, these laboratory changes occurred despite continued enteral nutritional support and a modest increase in body weight in all five patients during follow-up. Daily enteral energy intake ranged from 1450 to 1800 kcal, while body weight increased by 1.0–3.0 kg across participants. Although weight gain alone cannot establish adequate nutritional status, the absence of weight loss alongside continued enteral feeding provides additional clinical context for interpreting the observed decreases in serum proteins and selected micronutrient-related measurements during inflammatory episodes. These observations further support the need to interpret laboratory changes together with clinical and anthropometric findings rather than in isolation.

The observed patterns are biologically consistent with the established acute-phase response. During systemic inflammation, pro-inflammatory cytokines, particularly IL-6, TNF-α, and IL-1β, alter hepatic protein synthesis, increasing the production of positive acute-phase proteins while reducing negative acute-phase proteins such as albumin and transferrin [40,41]. Inflammatory activity may also affect serum Fe through hepcidin-mediated Fe sequestration and reduce circulating Zn through redistribution between plasma and tissues. These mechanisms provide a plausible explanation for the concurrent increase in inflammatory markers and decrease in several measured laboratory parameters observed during infectious episodes in the present cases [42]. Importantly, however, the current study was observational and was not designed to determine the relative contribution of inflammation, nutritional intake, catabolism, or other disease-related factors to individual laboratory values.

The present observations should be interpreted within, rather than in opposition to, contemporary nutritional assessment frameworks. GLIM recognizes inflammation or disease burden as an etiologic criterion for malnutrition, whereas the diagnosis itself requires the combination of etiologic and phenotypic criteria [43,44]. Laboratory inflammatory markers are not required as phenotypic criteria, nor does the present study propose their routine measurement for the diagnosis of malnutrition. Instead, the relevance of inflammatory assessment in the present case series concerns the interpretation of laboratory investigations when such measurements are clinically available [45].

This interpretation is also consistent with ASPEN and ESPEN recommendations, which caution against the use of serum albumin as an isolated marker of nutritional status because of its strong association with inflammatory activity [46,47]. Thus, the present findings should not be interpreted as supporting the reintroduction of albumin, transferrin, serum Fe, or other laboratory measurements as independent diagnostic criteria for malnutrition. Rather, the longitudinal trajectories observed in these patients illustrate why abnormal laboratory values obtained during inflammatory episodes require clinical context. In Patients A–C, for example, the lowest concentrations of several measured parameters frequently coincided with periods of increased inflammatory activity, whereas more stable profiles were observed in Patient D in the absence of documented infection.

The distinction between acute infectious episodes and periods without clinically evident infection was also informative. Acute inflammatory episodes were readily identifiable through clinical findings and marked changes in inflammatory biomarkers. However, the absence of an acute infection did not necessarily imply the absence of all inflammatory activity. Several patients had clinical conditions potentially associated with persistent inflammatory burden, including pressure injuries, prolonged immobility, recurrent infections, and chronic tissue injury. Accordingly, mildly abnormal laboratory values between infectious episodes should not automatically be attributed either to persistent malnutrition or to inflammation alone. Instead, longitudinal trends and the broader clinical context may provide more useful interpretative information than a single laboratory measurement [48].

Not all measured parameters demonstrated the same longitudinal pattern. Serum proteins, including albumin, transferrin, and total protein, showed pronounced fluctuations in association with infectious episodes, consistent with their known sensitivity to the acute-phase response. Micronutrient-related measurements require a different interpretative approach because the effects of inflammation vary according to the individual micronutrient [49]. In the present cases, serum Fe and Zn concentrations tended to decrease during infectious periods, whereas vitamin A, vitamin D, and folate concentrations showed comparatively limited fluctuations. According to the ESPEN guideline on micronutrients, inflammation may substantially alter circulating micronutrient concentrations through redistribution and other inflammation-related mechanisms, and low blood concentrations do not necessarily indicate true deficiency or depletion. ESPEN therefore recommends that the magnitude of the inflammatory response be considered when interpreting micronutrient measurements and that CRP be determined concurrently with micronutrient analysis [50]. Given the small sample size of the present case series, the comparatively stable vitamin concentrations observed in our patients should not be interpreted as evidence that these vitamins are unaffected by inflammation or as support for their use as alternative markers of nutritional status.

It should also be emphasized that the measurement of individual micronutrient concentrations is not proposed here as a routine component of nutritional assessment for all patients. According to ESPEN guidance, laboratory assessment of specific micronutrients is particularly relevant in patients with clinical conditions, nutritional support modalities, suspected deficiencies, or other risk factors that justify targeted investigation [50]. In the present case series, micronutrient measurements were used to characterize longitudinal laboratory changes in a highly selected population receiving long-term enteral nutrition rather than to suggest their routine application in general clinical practice.

These case-based observations formed the basis for the proposed conceptual framework. The framework is not intended to replace GLIM, ESPEN, ASPEN, or established nutritional screening and diagnostic approaches. Nor does it propose laboratory or inflammatory biomarkers as diagnostic criteria for malnutrition. Its purpose is narrower: to provide a structured approach for considering inflammatory activity when interpreting laboratory parameters that are already available in clinical practice.

Within this framework, the first consideration is whether there is evidence of active inflammatory activity based on the available clinical and laboratory information. During active inflammation, concentrations of albumin, transferrin, total protein, serum Fe, and Zn may be substantially influenced by the inflammatory response and should therefore be interpreted cautiously. These findings should be considered alongside clinical findings, longitudinal trends, and established phenotypic and etiologic criteria rather than used independently to determine nutritional status.

**Figure 3:**
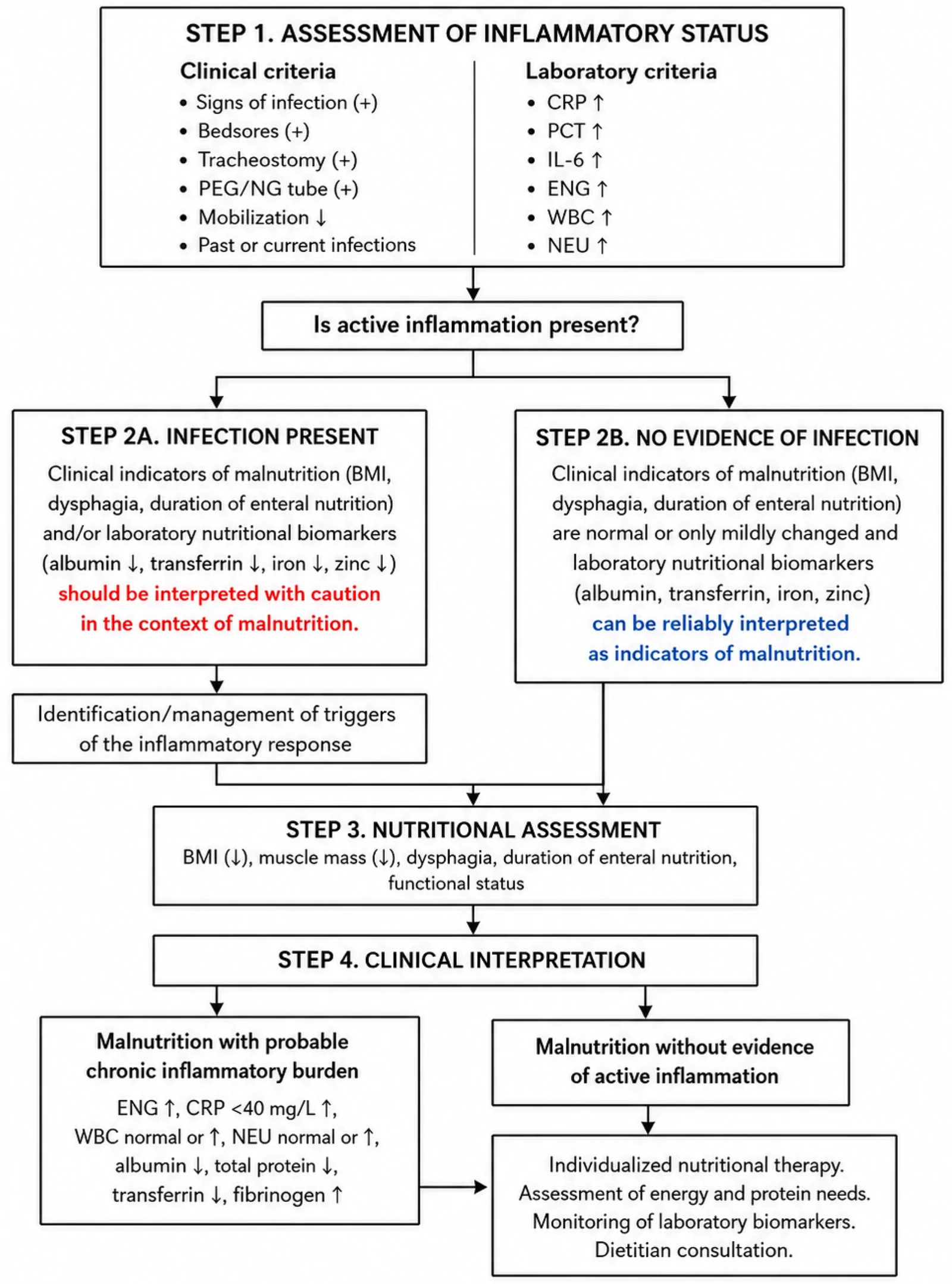
Proposed conceptual framework for interpreting laboratory biomarkers in the context of inflammatory activity.

In clinically stable periods without evidence of active inflammation, the influence of the acute-phase response may be reduced (Figure 3); however, these laboratory parameters should still not be regarded as standalone diagnostic indicators of malnutrition. Their interpretation should remain integrated with established clinical, phenotypic, and anthropometric criteria. This distinction is particularly relevant in patients with advanced neurological diseases, in whom nutritional impairment and inflammatory conditions frequently coexist [51].

The practical implication of this approach is therefore not to increase the role of laboratory testing in the diagnosis of malnutrition, but to reduce the risk of overinterpreting abnormal laboratory results. A decrease in albumin or transferrin during an infectious episode should not, by itself, be assumed to demonstrate deterioration in nutritional status or inadequacy of enteral nutrition. Similarly, improvement in these parameters following resolution of inflammatory activity should not necessarily be interpreted as evidence of nutritional recovery. Their clinical meaning depends on the circumstances in which they were measured and should be integrated with established nutritional assessment methods.

### Study Limitations

Several limitations should be considered when interpreting these findings. The study included only five patients from a single palliative care facility, which substantially limits generalizability and precludes meaningful inferential statistical analysis. However, the purpose of this prospective longitudinal case series was not to estimate population-level effects or establish diagnostic thresholds, but to characterize within-patient temporal patterns across repeated measurements. Each participant was followed longitudinally across 23 predefined assessment time points, allowing inflammatory and laboratory changes to be examined within the clinical course of individual patients. Thus, although the sample size is insufficient for statistical inference, the repeated-measures case-based design provides detailed exploratory evidence regarding temporal associations that can inform hypothesis generation and the development of the proposed conceptual framework.

The descriptive case-series design does not permit causal inference or determination of diagnostic thresholds, sensitivity, specificity, or predictive value for any of the measured laboratory parameters. Furthermore, the heterogeneous underlying neurological diseases and clinical courses may have independently influenced both inflammatory and metabolic parameters. The requirement for complete longitudinal follow-up resulted in the exclusion of 18 of the 23 initially enrolled patients from the final analysis, which may have introduced attrition and survivorship bias, as patients who died or left the study facility before completion of follow-up were not represented in the final longitudinal analysis. Accordingly, the findings and the proposed framework should be considered exploratory and hypothesis-generating and require prospective evaluation in larger and more diverse patient populations.

## 5. Conclusions

The present longitudinal case series demonstrated that changes in several laboratory parameters related to nutritional and metabolic status were temporally associated with inflammatory activity in multimorbid patients with advanced neurological diseases receiving long-term enteral nutrition. Decreases in albumin, transferrin, total protein, serum Fe, and Zn frequently coincided with increased inflammatory activity during infectious episodes, whereas vitamin A, vitamin D, and folate showed comparatively limited fluctuations. Importantly, these laboratory changes occurred despite continued enteral nutritional support and modest increases in body weight in all participants, highlighting the importance of interpreting laboratory findings together with inflammatory, clinical, and anthropometric information.

Based on these case-based observations and current evidence, a conceptual framework was proposed to support the interpretation of laboratory parameters in the context of inflammatory activity. The framework is intended as an interpretative aid rather than as an alternative diagnostic system for malnutrition and does not support the use of laboratory biomarkers as standalone diagnostic criteria. Given the small sample size and descriptive case-series design, the proposed framework should be considered hypothesis-generating and requires prospective validation in larger and more diverse patient populations.

Future research should prospectively evaluate the observed longitudinal relationships in larger and more diverse patient populations and determine whether similar patterns are observed across different neurological conditions and nutritional support settings. Further studies should also assess the clinical applicability and reproducibility of the proposed conceptual framework and examine whether integrating longitudinal inflammatory, clinical, anthropometric, and laboratory information improves the interpretation of laboratory changes compared with isolated measurements.

## Figures and Tables

**Table 1:** Demographic and clinical characteristics of the study participants.

Characteristic	A	B	C	D	E
Sex	Male	Female	Male	Female	Female
Age (years)	28	79	82	18	44
Baseline weight (kg)	40	51	47	23	59
Height (cm)	171	178	173	132	183
Primary diagnosis	MPAN	Ischemic stroke	Parkinson’s disease	Cerebral palsy	Huntington’s disease
Respiratory support	Tracheostomy	Spontaneous breathing	Spontaneous breathing	Spontaneous breathing	Spontaneous breathing
Enteral feeding route	PEG	NG tube	PEG	PEG	NG tube
Duration of enteral nutrition (months)	5	4	2	7	9
Enteral nutrition before hospitalization	Yes	No	No	Yes	Yes
Baseline BMI (kg/m^2^)	13.7	16.1	15.7	13.2	17.6
Enteral energy intake (kcal/day)	1450	1650	1550	1500	1800
Final weight (kg)	42.3	52	49	26	60
Weight change during follow-up (kg)	+2.3	+1	+2	+3	+1
Duration of current hospitalization (months)	5	9	2	1	2
Duration of palliative care (months)	27	4	18	9	10

Abbreviations: MPAN, mitochondrial membrane protein-associated neurodegeneration; PEG, percutaneous endoscopic gastrostomy; NG, nasogastric tube.

**Table 2:** Clinical characteristics associated with the risk of malnutrition and inflammatory burden among the study participants.

Clinical Characteristic	A	B	C	D	E
Advanced neurological disease	Yes	Yes	Yes	Yes	Yes
Severe neurological impairment	Yes	Yes	Yes	Yes	Yes
Dysphagia	Yes	Yes	Yes	Yes	Yes
Inadequate food and fluid intake before the current hospitalization	Yes	Yes	Yes	Yes	Yes
Low body mass index (BMI)	Yes	Yes	Yes	Yes	Yes
Body mass index (kg/m^2^)	13.7	16.1	15.7	13.2	17.6
Sarcopenia	Yes	Yes	Yes	Yes	No
Bedridden status or markedly reduced physical activity	Yes	Yes	Yes	Yes	Yes
Multimorbidity	Yes	Yes	Yes	Yes	Yes
Acute and/or chronic inflammation	Yes	Yes	Yes	No	No
Recurrent infections	Yes	Yes	Yes	No	No
Number of infectious episodes during the previous 3 months	4	2	2	–	1
Infection confirmed by	Clinical + microbiological	Clinical	Clinical + microbiological	–	Clinical
Pressure injuries	Yes	Yes	Yes	No	Yes
Malabsorption and/or gastrointestinal disorders	Yes	Yes	Yes	Yes	Yes
Chronic heart failure	No	Yes	Yes	No	No
Chronic kidney disease	No	No	Yes	No	No
Chronic obstructive pulmonary disease	No	No	No	No	No
Liver disease	No	No	No	No	No
Dementia and cognitive impairment	Yes	Yes	Yes	Yes	Yes
Other psychiatric disorders	Yes	Yes	Yes	Yes	Yes
Polypharmacy	Yes	Yes	Yes	Yes	Yes

Abbreviations: BMI, body mass index.

**Table 3:** Inflammatory biomarker values during infectious and non-infectious periods (minimum–maximum values).

Biomarker	A		B		C		D		E	
	Y	N	Y	N	Y	N	Y	N	Y	N
CRP, mg/L	16.55–274.67	<10	39.24–111.40	<10	44.61–174.55	<10	–	<10	10.81–25.13	<10
Procalcitonin, ng/mL	0.091–1.450	<0.05	0.094–0.098	<0.05	0.141–2.82	<0.05	–	<0.05	0.041–0.069	<0.05
Leukocyte count (×10^9^/L)	10.46–12.15	4.78–8.47	12.51–15.34	5.90–7.84	9.75–11.70	7.12–7.91	–	5.47–5.88	8.01–11.27	6.74–7.21
Neutrophils (%)	74.8–77.7	54.1–68.2	77.7–86.6	51.7–65.5	72.4–77.7	60.6–70.1	–	48.5–51.8	68.4–71.9	65.2–69.3
Absolute neutrophil count (×10^9^/L)	7.52–9.44	2.77–5.06	10.83–12.70	2.80–6.06	7.06–9.03	4.79–4.99	–	2.65–3.05	5.48–6.89	4.39–5.01
ESR, mm/h	49–68	28–41	70–82	43–68	66–73	22–38	–	4–19	21–47	20–38
IL-6, pg/mL	19.5–120.0	1.1–2.9	21.8–41.2	5.7–8.4	23.3–26.2	1.8–2.7	–	2.6–3.9	11.3–16.8	1.9–3.2
TNF-α, pg/mL	9.3–11.0	6.8	3.1–7.9	4.8–5.9	2.8–7.9	3.2–4.1	–	4.6	3.9–8.1	1.4–3.9
Hepcidin, ng/mL	19.2–15.9	11.5	29.3–24.7	9.12	22.7–21.9	13.6	–	7.6	24.3–18.7	6.8

Abbreviations: Y, periods of confirmed infection (clinical and/or microbiological); N, periods without clinical evidence of infection.

**Table 4:** Laboratory biomarkers related to nutritional and metabolic status during infectious and non-infectious periods (minimum–maximum values).

Biomarker	A		B		C		D		E	
	Y	N	Y	N	Y	N	Y	N	Y	N
Serum proteins										
Albumin, g/L	23.6–26.2	27.6–28.8	19.1–23.3	22.6–31.2	20.5–27.3	31.4–41.2	–	44.5–51.2	35.7–38.2	36.1–41.8
Total protein, g/L	53.0–55.4	59.2–59.3	55.1–65.6	54.8–69.7	52.4–58.2	56.8–61.4	–	74.7–83.4	67.8–68.1	68.4–71.1
Transferrin, g/L	0.87–1.16	1.4–2.3	1.11–1.18	1.34–1.98	0.95–1.10	1.31–1.54	–	3.74–3.77	2.02–2.09	2.36–2.59
Micronutrient-related measurements										
Se, µg/L	64	66	64	68	54	61	–	63	96	102
Serum Fe, µmol/L	2.34–5.94	3.86–5.99	2.75–9.04	3.68–10.66	5.86–29.71	27.55–33.60	–	57.65–66.30	12.5	13.1
Zn, µmol/L	3.93–10.55	10.94–11.40	6.07–8.86	7.64–9.09	8.71–10.30	9.11–10.60	–	13.91–14.02	16.3	17.6
Vitamins										
Vitamin A, µmol/L	1.45	1.51	0.61	0.73	1.46	1.41	–	1.63	1.59	1.61
Vitamin D, nmol/L	108	111	92.4	96.8	177	191	–	141	154	155
Folate (Vitamin B9), nmol/L	4.8	6.3	6.0–11.2	11.7	12.4	13.5	–	15.9	15.5	16.1

Abbreviations: Y, periods of confirmed infection; N, periods without clinical evidence of infection. Serum proteins, micronutrient-related measurements, and vitamins are presented separately because their biological responses to inflammation and limitations of interpretation differ.

## Data Availability

Data supporting reported results can be found at https://www.midas.lt:443/action/resources/1cd0dc40-29aa-4c2c-b545-9a36616418e9 (accessed on 12 June 2026).

## Abbreviations

The following abbreviations are used in this manuscript:

ASPEN	American Society for Parenteral and Enteral Nutrition
BMI	Body mass index
CRP	C-reactive protein
EPUAP	European Pressure Ulcer Advisory Panel
ESR	Erythrocyte sedimentation rate
ESPEN	European Society for Clinical Nutrition and Metabolism
Fe	Iron
GDPR	General Data Protection Regulation
GLIM	Global Leadership Initiative on Malnutrition
IL-1β	Interleukin-1 beta
IL-6	Interleukin-6
MPAN	Mitochondrial membrane protein-associated neurodegeneration
NG	Nasogastric
NPIAP	National Pressure Injury Advisory Panel
PEG	Percutaneous endoscopic gastrostomy
Se	Selenium
TNF-α	Tumor necrosis factor alpha
Zn	Zinc
